# *Bacillus subtilis* M2-Fermented Soybean Meal Improves Growth Performance and Modulates Cecal Microbiota in Dongxiang Green-Shell Laying-Type Chicken

**DOI:** 10.3390/microorganisms14071589

**Published:** 2026-07-21

**Authors:** Huifang Lu, Lidan Zhou, Enchao Yu, Xinru Cai, Jinhua Zhang, Qiufen Li, Baosheng Liu

**Affiliations:** 1 College of Animal Science and Technology, Jiangxi Agricultural University, No. 1101, Zhimin Avenue, Nanchang 330045, China; 2 School of Animal Science, Jiangxi Agricultural Engineering Vocational College, No. 266, Site Avenue, Zhangshu 331200, China

**Keywords:** *Bacillus subtilis* M2, fermented soybean meal, growth performance, cecal microbiota, chicken

## Abstract

Feeding animals with soybean meal (SBM) fermented by probiotics normally confers benefits. This study evaluated the growth-promoting effects of SBM fermented with *Bacillus subtilis* M2 (M2-FSBM), a probiotic strain with antimicrobial activity, as a replacement for conventional SBM in chicken diets. A total of 264 one-day-old Dongxiang green-shell laying-type chickens were randomly assigned to four groups (six replicates/group, 11 birds/replicate) and fed a basal diet with 0% (control, with 35% SBM), 25%, 50%, or 75% of SBM replaced by M2-FSBM for 45 days. Growth performance, feed consumption, serum insulin-like growth factor and epidermal growth factor, small intestinal morphology, and cecal microbiota diversity were evaluated. Compared with the control, M2-FSBM replacement significantly increased body weight at day 15 (all replacement levels) and day 30 (50% and 75% groups), and at day 45 (75% group) (*p* < 0.05). The improved growth was attributed to increased average daily feed intake rather than improved feed conversion ratio. The 75% M2-FSBM group showed significantly increased villus height in the duodenum, jejunum, and ileum, and elevated jejunal villus height-to-crypt depth ratio (*p* < 0.05). Cecal microbiota analysis revealed increased abundances of *Faecalibacterium* and *Negativibacillus* and decreased abundances of *Mediterraneibacter*, *Blautia*, and *unclassified_f_Lachnospiraceae*, while *Lactobacillus* and *Limosilactobacillus* remained stable. Replacing SBM with M2-FSBM improves growth performance in young chickens, likely through increased feed intake and improved intestinal morphology, accompanied by beneficial modulation of cecal microbiota. M2-FSBM shows promise as a growth-promoting alternative feed material for poultry.

## 1. Introduction

Soybean meal (SBM), a by-product of soybean oil extraction, is the most commonly used and an important protein source in animal feed formulation. Studies have shown that SBM contains antinutritional factors, including antigenic proteins (e.g., glycinin and β-conglycinin), trypsin inhibitors, oligosaccharides, and phytic acid [1], which can damage the intestinal structure of animals and impair the digestion and absorption of nutrients, particularly protein utilization in young animals [2]. Microbial fermentation is a traditional and economically viable feed processing technology [3]. It significantly reduces the levels of antinutritional factors in SBM, increases the content of small peptides and low-molecular-weight proteins [4], and generates peptide compounds and metabolites with probiotic properties [5,6]. Consequently, microbial fermentation optimizes the composition of the gut microbiota, improves intestinal morphology, and enhances feed quality and digestibility [7].

*Bacillus* spp. are among the earliest discovered bacteria. They exhibit remarkable resistance to environmental stress, rapid growth and reproduction, and the ability to secrete various extracellular antimicrobial substances and hydrolases [8]. *Bacillus* probiotics exert their beneficial functions through mechanisms including modulation of the animal gut microbiota structure, antagonism against pathogenic bacteria, and enhancement of feed conversion efficiency [9]. Based on extensive experimental research and practical evidence in livestock and poultry applications, *B. subtilis* has been recognized as an alternative to antibiotics [10]. Seo et al. employed two-dimensional gel electrophoresis coupled with mass spectrometry to demonstrate that *B. subtilis* degrades macromolecular proteins into small peptides during SBM fermentation [11]. Specifically, *B. subtilis* KCCM11438P fermentation of SBM for 24 h degraded 42% of glycinin, 82.8% of β-conglycinin, and 66% of trypsin inhibitors present in the SBM. Furthermore, Irawan et al. reported that replacing SBM with fermented SBM (FSBM) in diets improved final body weight (BW) of broilers, especially in the starter period whereas the effects on average daily gain (ADG) and feed conversion ratio (FCR) were mostly dependent on microbial strains used for fermentation [12].

Previous studies on feeding FSBM to animals have predominantly utilized probiotic strains isolated from environmental sources, with functionalities such as fibrolytic activity, enzyme production, or acid generation. However, the use of endogenous probiotic strains derived from the animal intestine, capable of producing antimicrobial metabolites, for SBM fermentation has not been previously reported. In this study, we used *B. subtilis* M2, an intestinal endogenous strain isolated from the gut of healthy pigs [13], which produces lipopeptide antimicrobial complexes with broad-spectrum activity under both aerobic and anaerobic conditions (unpublished data), to ferment conventional SBM. The FSBM was then used to replace SBM in chicken diets. Growth performance, feed consumption, serum growth-related factors, intestinal morphology, and gut microbiota diversity were evaluated to assess the growth-promoting effects of *B. subtilis* M2-fermented SBM (M2-FSBM) on chickens. This work aims to provide a foundation for developing the M2 strain as a growth-promoter probiotic in animals and to offer insights for developing other anti-pathogen probiotic strains with disease-preventing and growth-promoting properties.

## 2. Materials and Methods

### 2.1. SBM and Its Pretreatment

SBM used in this study was purchased from a small-scale feed manufacturer located in Jiangxi Province, China, with the soybeans originating from Jiangsu Province, China. The SBM was ground to pass through a 45-mesh sieve. The resulting SBM powder (SBMP) was collected and stored at ambient temperature for the entire duration of the experiments. For experimental use, aliquots of 10 g SBMP were weighed into 50 mL screw-capped conical flasks. The SBMPs were subsequently autoclaved at 121 °C for 20 min and allowed to cool naturally to room temperature prior to use. Multiple batches of sterilized SBMP were prepared following this procedure for each experimental replicate.

### 2.2. Preparation of M2-FSBM

An aliquot of sterilized SBMP was transferred to a resealable plastic bag. Water was added at a liquid-to-solid ratio of 3:5 (*v*/*w*, water:SBMP) under aseptic conditions. *B. subtilis* M2 seed culture, equivalent to 5% (*v*/*w*, seed:SBMP) of the SBMP, was inoculated and thoroughly mixed. The inoculated mixture was incubated at 37 °C for 12 h to produce the primary FSBM starter culture. The quality of the starter culture was assessed based on visual characteristics and viable bacterial count to ensure consistency.

For the main fermentation, SBM was inoculated with the prepared starter culture at a ratio of 9:1 (*w*/*w*, SBM:starter). Following adding water at 60% (*v*/*w*) of the dry matter basis of the SBM and thorough mixing, the mixture was fermented for 24 h to yield the final M2-FSBM product.

### 2.3. Nutrient Content Analysis of SBM and M2-FSBM

Samples from three consecutive batches of M2-FSBM were collected both before and after the fermentation process. For each batch and time point, two replicate samples were obtained. The contents of crude protein, crude fat, crude ash, and crude fiber in the samples were determined in accordance with the following Chinese National Standards: GB/T 6432-2018 [14], GB/T 6433-2006 [15], GB/T 6438-2007 [16], and GB/T 6434-2022 [17], respectively. 

### 2.4. Experimental Design and Animal Management

A total of 264 one-day-old Dongxiang green-shell laying-type chickens (♂) were randomly assigned to four groups (Con, FSBM25, FSBM50 and FSBM75) based on their BW, with six replicates per group and 11 birds per replicate. All birds within the same replicate were housed in a single cage as an experimental unit. Group 1 served as the control group (Con), receiving a basal diet without M2-FSBM substitution. Groups 2 (FSBM25), 3 (FSBM50), and 4 (FSBM75) received experimental diets in which SBM in the basal diet was replaced with M2-FSBM at levels of 25%, 50%, and 75% (on a dry matter basis), respectively (Table 1). All procedures in the experiment were reviewed and approved by the Experimental Animal Ethics Committee of Jiangxi Agricultural University (approval No.: JXAULL-12-05-2024).

### 2.5. Diet Formulation and Preparation

A conventional corn–SBM diet was formulated according to the nutrient requirements for growing laying hens (0–8 weeks of age) specified in the Chinese Ministry of Agriculture’s Feeding Standard of Chicken (NY/T 33-2004 [18]). Throughout the experimental period, all feed ingredients (with the exception of SBM) were processed in a single batch. At feeding time, the freshly prepared M2-FSBM was blended with SBM at the designated ratios. This mixture was then thoroughly mixed in the specified proportions with the pre-processed basal feed components. The M2-FSBM was prepared immediately before blending, and all the prepared diets were fed exclusively on the day of preparation.

### 2.6. Bird Immunization and Husbandry Management

The poultry house was fumigated with potassium permanganate–formaldehyde solution prior to chicken arrival following standard disinfection protocols. Chickens received Marek’s disease vaccination at 1 day of age, followed by drinking water vaccination with Newcastle disease-infectious bronchitis bivalent live vaccine (La Sota strain + H120 strain) at 7 and 14 days of age. Throughout the trial, birds had ad libitum access to feed and water. Freshly prepared feed was provided daily with precise recording of both feed provision and residual quantities per replicate. Ambient temperature was maintained at 37 °C on day 1, then reduced by 2–3 °C weekly until reaching 25–26 °C which was held constant thereafter. Relative humidity was controlled at 50% to 60%.

### 2.7. Growth Performance Measurement

Growth performance was assessed on days 15, 30 and 45. Birds within each replicate were fasted overnight with free access to water prior to weighing. Meanwhile, the residual feed in each replicate feeder was weighed to determine feed consumption during the preceding interval. ADG, average daily feed intake (ADFI), and FCR were calculated based on the recorded BWs and feed consumption for each replicate.

### 2.8. Sample Collection

On days 15, 30, and 45, one chicken per replicate closest to the mean body weight was selected and euthanized. Blood was collected via cardiac puncture to prepare serum for subsequent growth factor determination. On day 30, segments from the middle duodenum, jejunum, and ileum were collected from chickens in groups 1 and 4. These tissues were fixed in 4% paraformaldehyde and subsequently submitted to Wuhan Servicebio Technology Co., Ltd. (Wuhan, China), for paraffin sectioning (5 μm thickness) and hematoxylin–eosin (HE) staining. Concurrently, cecal contents were collected, rapidly frozen in liquid nitrogen, and shipped to Guangdong Magigene Biotechnology Co., Ltd. (Guangzhou, China), for gut microbiota diversity analysis.

### 2.9. Serum Growth Factor Assay

Serum concentrations of epidermal growth factor (EGF) and insulin-like growth factor (IGF) were quantified using chicken-specific ELISA kits (Cat# MM-050901 and MM-6071401 for EGF and IGF respectively, Jiangsu Meimian Industrial Co., Ltd., Yancheng, China) following the manufacturer’s protocols.

### 2.10. Intestinal Villus Height and Villus-to-Crypt Ratio Measurement

Representative tissue sections from each intestinal segment per bird were selected. Villus height (VH) and crypt depth (CD) were measured in a blinded manner using an Olympus BX53 biological microscope (Olympus Corporation, Tokyo, Japan) with ImageJ software (v1.54 g/Java1.8.0_345). For each section, five intact villi were measured for height, and the adjacent crypts were measured for depth. The mean for each section was calculated and recorded as the VH and CD for the corresponding bird. The VH-to-CD ratio (VH/CD) was then computed. The final results are presented as the mean from six biological replicates per group.

### 2.11. Cecal Microbial Diversity Analysis

Cecal microbiota DNA was extracted and the V3-V4 region of the 16S rRNA gene was sequenced (Illumina NovaSeq, Illumina, Inc., San Diego, CA, USA; paired-end 250 bp). Sequences were processed with QIIME2 (v2020.11.0). The amplicon sequence variants (ASVs) were identified using DADA2 and bacterial taxonomy was assigned against the SILVA 138 database at default settings. Based on the ASV results, α- and β-diversity of cecal microbiota were compared between groups 1 and 4. Microbial taxonomic diversity was statistically evaluated at both the phylum and genus levels.

### 2.12. Statistical Analysis

Data were analyzed using SPSS 27.0 (IBM Corp., Armonk, NY, USA). One-way ANOVA was performed to assess overall group differences. Where significant effects (*p* < 0.05) were detected by ANOVA, post hoc pairwise comparisons between groups were conducted using the Least Significant Difference (LSD) test. Alpha diversity analysis of the gut microbiota was performed using R software (v3.5.1). Intergroup differences were assessed using Student’s *t*-test and Wilcoxon rank-sum test for parametric and non-parametric comparisons, respectively.

## 3. Results

### 3.1. Physical Properties and Viable Count of Starter Culture

Upon completion of fermentation, the starter culture exhibited distinct phenotypic changes: its color transitioned from pale yellow to yellowish-brown, while the aroma shifted from the original mild SBM scent to a breadcrumb-like fragrance. The texture became friable, compacting into a loose mass under gentle hand pressure yet disintegrating readily upon light finger contact. Repeated experiments confirmed that these physical characteristics represent the typical phenotypic profile of fully fermented starter culture and the viable cell counts of *B. subtilis* M2 stabilized at 1.1 × 10^9^ CFU/g.

### 3.2. Nutrient Content Determination

Compared to SBM, M2-FSBM exhibited significantly increased crude protein and crude ash content (*p* < 0.05). Conversely, no significant changes (*p* > 0.05) were observed in crude fat or crude fiber content (Table 2).

### 3.3. Growth Performance of the Birds

At day 15, chickens in all M2-FSBM replacement groups exhibited significantly higher BWs (*p* < 0.05) compared to the control group (Table 3). By day 30, the low-replacement group (FSBM25) showed no significant difference in BW relative to the control (*p* > 0.05), whereas significantly greater BWs persisted in the medium- (FSBM50) and high-replacement (FSBM75) groups (*p* < 0.05). At day 45, only the high-replacement group (FSBM75) maintained a statistically significant increase in BW over the control (*p* < 0.05).

### 3.4. Feed Conversion Ratio Calculation

To elucidate the underlying reasons for the growth-promoting effects of substituting SBM with M2-FSBM observed in this study, we further analyzed the FCR of the experimental chickens across three distinct phases. As shown in Table 3, substituting SBM with M2-FSBM significantly increased the ADFI of the chickens (*p* < 0.05; using days 1–15 as an example, as the significant differences in BW among groups beyond 15 days of age markedly influenced the comparability of feed intake data). However, FCR did not differ significantly among any of the groups throughout the trial (*p* > 0.05). These results indicate that while dietary inclusion of M2-FSBM promotes the growth of young chicks, this growth promotion is primarily achieved through increased feed intake rather than improved feed conversion efficiency.

### 3.5. Serum Growth Factor Analysis

Serum concentrations of IGF and EGF in chickens at days 15, 30, and 45 were determined using a sandwich enzyme-linked immunosorbent assay (ELISA) with dual antibodies. Significant differences (*p* < 0.05) in the concentrations of both IGF and EGF were observed between some M2-FSBM substitution groups and the control group within the same growth period (Supplementary Table S1). However, these significant changes were inconsistent with the alterations in chicken’s BW gain. Therefore, it is suggested that the increased growth rate observed in chickens fed diets containing M2-FSBM as a replacement for SBM was not mediated through changes in circulating IGF or EGF concentrations.

### 3.6. Villus Height and Villus-to-Crypt Ratio

Based on the aforementioned analysis of chicken growth performance, the substitution of 75% SBM in the diet with M2-FSBM yielded the most pronounced growth-promoting effect and days 15–30 represented the mid-term of the experiment. Consequently, subsequent histological examination of the small intestine and analysis of cecal microbiota were exclusively conducted using samples from the control group and the 75% M2-FSBM substitution group at 30 days of age. Compared with the control group, dietary replacement with 75% M2-FSBM significantly increased VH in the duodenum, jejunum, and ileum (*p* < 0.05, Table 4). No significant difference was observed in CD across these intestinal segments (*p* > 0.05). Furthermore, the VH/CD in the jejunum was significantly elevated (*p* < 0.05).

### 3.7. Cecal Microbial Diversity of the Birds

**Figure 1:**
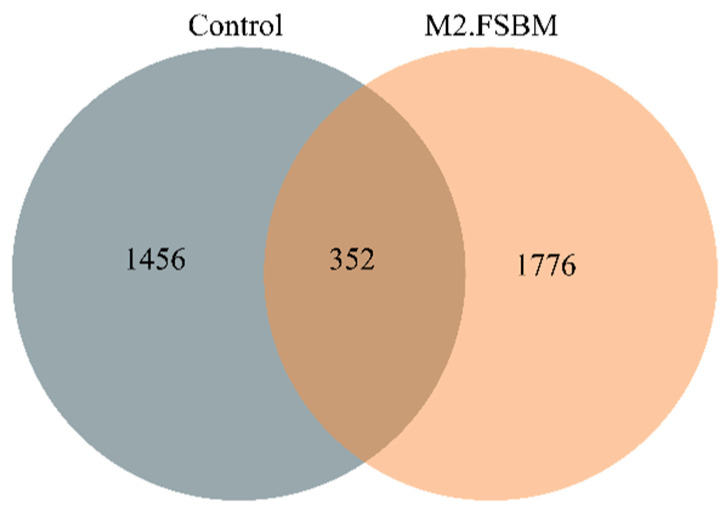
Venn diagram of cecal microbiota. Note: Control, control group; M2.FSBM, 75% M2-FSBM substitution group.

*Sequencing results.* A total of 3584 valid ASVs were acquired. Among these, 352 ASVs were shared between the control group and the 75% M2-FSBM substitution group, while the control group and 75% M2-FSBM substitution group harbored 1456 and 1776 unique ASVs respectively (Figure 1).

**Figure 2:**
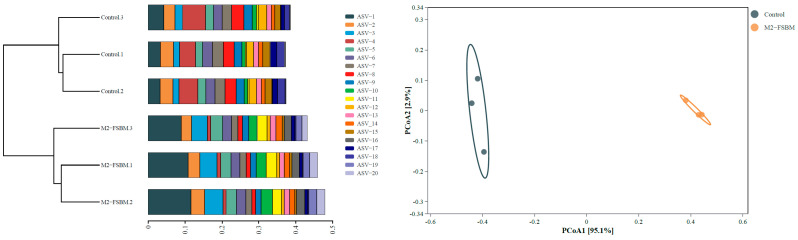
Beta diversity analysis of microbial composition in the cecum of chickens. (**Left**) Sample cluster analysis of the top 20 ASVs in the cecal microbiota. (**Right**) PCoA analysis of cecal microbiota β-diversity in chickens. Note: control.1 to 3 and M2-FSBM.1 to 3 represent the results of the control group and the FSBM75 group respectively. The ellipses in the PCoA plot represent the 95% confidence intervals for different groups.

*Effects of M2-FSBM on the cecal microbial diversity.* The alpha diversity analysis revealed that the 75% M2-FSBM substitution group showed no significant differences (*p* > 0.05) in the Chao1, ACE or Shannon indices compared to the control group (Table 5). However, the Simpson index was significantly increased (*p* < 0.05). This result indicates that feeding chickens M2-FSBM did not markedly affect microbial abundance in the cecum, but significantly enhanced the diversity of the microbial community (*p* < 0.05). To further explore the compositional diversity of different taxa within the gut microbiota, β-diversity analyses of microbial community structure between the two groups were conducted (Figure 2). The sample clustering analysis revealed that the three replicates from both the control group and the 75% M2-FSBM replacement group clustered separately, forming two distinct clades. The relative abundance of identical ASVs was similar within each clade but markedly different between clades. Similarly, the principal coordinate analysis (PCoA) results clearly separated the control and the FSBM75 group in the ordination space, indicating significant differences in their microbial community compositions. Collectively, the α- and β-diversity analyses demonstrate that replacing SBM with M2-FSBM in the diet alters the relative abundances of various taxa within the cecal microbial community, thereby influencing the structural diversity of the cecal microbiota.

*Analysis of gut microbiota composition at different taxonomic levels*. To further investigate the impact of replacing SBM with M2-FSBM on the gut microbial diversity in chickens, we analyzed the relative abundance of cecal microbiota at both phylum and genus levels. As shown in Table 6, the cecal microbiota in both groups was predominantly composed of Bacillota, Pseudomonadota and Actinomycetota. Bacillota served as the overwhelmingly dominant phylum, accounting for approximately 98% of the total microbiota, with no significant difference observed between the trial and control groups (*p* > 0.05).

At the genus level, compared with the control group, the FSBM75 group exhibited significantly increased abundances of *Faecalibacterium* and *Incertae_Sedis* (*p* < 0.05, Table 7), while the abundances of *Mediterraneibacter*, *Blautia*, and *unclassified_f_Lachnospiraceae* were significantly decreased (*p* < 0.05). In contrast, the abundances of *Lactobacillus*, *unclassified_f_Ruminococcaceae*, *Limosilactobacillus* and *Negativibacillus* remained relatively stable (*p* > 0.05).

## 4. Discussion

*Bacillus subtilis* exhibits remarkable environmental adaptability. Endogenous *B. subtilis* strains within the animal gastrointestinal tract competitively inhibit various transient pathogenic bacteria, including *Staphylococcus aureus*, *Clostridium perfringens*, and *Escherichia coli* [19]. Furthermore, the probiotic mechanisms of *B. subtilis* encompass maintaining intestinal microecological balance, enhancing host immunity, improving antioxidative capacity [20,21], reducing populations of enteric pathogens while increasing beneficial microbiota, and elevating the activity of digestive enzymes in the host intestine [22]. Notably, it may serve as a feed additive alternative to antibiotics for improving growth performance in livestock and poultry [23]. Nevertheless, the safe utilization of *B. subtilis* remains a matter that requires unwavering attention from the scientific community [24]. The *B. subtilis* M2 strain, isolated from porcine intestine, demonstrates typical probiotic attributes as established in prior studies [13]. Critically, it secretes lipopeptide complexes with broad-spectrum antimicrobial activity, exhibiting potent inhibitory effects against *Staphylococcus aureus*, *Escherichia coli* K88, and *Salmonella* spp. These properties designate *B. subtilis* M2 as a promising therapeutic probiotic strain for development.

Poultry animals possess a relatively short digestive tract, which often limits the complete digestion and absorption of dietary nutrients. Microbial fermentation of feed offers a miraculous strategy to enhance feed digestibility and improve biological value, thereby promoting growth performance in chickens while concurrently mitigating environmental pollution from animal feces [25]. Studies indicate that microbial fermentation effectively degrades ANFs in feed ingredients such as SBM and corn gluten meal—including trypsin inhibitors and phytate—liberating key nutrients like amino acids and small peptides for more efficient absorption [26,27]. This process consequently elevates the digestibility of both protein and energy within the feed. In our study, replacing SBM with M2-FSBM in the diet significantly increased feed intake and protein consumption, thereby improving the growth performance of young chickens [28,29]. Moreover, the beneficial effects were more pronounced in the early phase compared to the later phase [12], and higher replacement ratios yielded better outcomes than lower ones.

The small intestine serves as the primary site for nutrient digestion and absorption in animals. VH and the jejunal VH/CD are critical indicators of intestinal digestive and absorptive capacity. Studies have shown that fermented feed significantly enhances villus development and jejunal VH/CD in chickens [30]. The underlying mechanism involves organic acids (e.g., lactic acid, acetic acid) produced during feed fermentation. These compounds reduce intestinal pH, suppress pathogenic bacteria proliferation, and promote colonization by beneficial microbiota such as *Lactobacillus* [31]. Consequently, mucosal damage caused by harmful bacteria is mitigated, while epithelial cell proliferation is stimulated, collectively fostering villus maturation. In the present study, the FSBM75 replacement group exhibited significantly higher VH (*p* < 0.05) in the duodenum, jejunum, and ileum compared to the control group. Concurrently, jejunal VH/CD was markedly elevated (*p* < 0.05), indicating that M2-FSBM effectively enhanced intestinal health and nutrient assimilation capacity in chickens, thereby promoting their growth performance [32].

The intestinal microbiota plays a pivotal role in host nutrient metabolism, immune regulation, and growth development. Studies have demonstrated that feeding fermented feed can significantly alter the gut microbial structure in broiler chicks, optimizing microbial balance [33]. *B. subtilis* M2 exhibits broad-spectrum antibacterial activity, and feeding M2-SBM inevitably induces changes in the gut microbiota composition of animals. According to the results of this study, the diversity of microbial species and their relative abundance in the cecum of chickens were markedly modified after feeding the diet with M2-FSBM. Interestingly, the genera (*Faecalibacterium* and *Incertae_Sedis*) that increased significantly mainly belong to the family Ruminococcaceae, while the three genera (*Mediterraneibacter*, *Blautia* and *unclassified_f_Lachnospiraceae*) that decreased significantly are all members of the family Lachnospiraceae. Further statistical analysis at the family level corroborated this trend (Supplementary Table S2): compared with the control group, the FSBM75 group showed a significant increase in Ruminococcaceae and a significant decrease in Lachnospiraceae, while Lactobacillaceae remained relatively stable. It should be noted, however, that the sample size for gut microbiota analysis in this study was relatively small, which may to some extent limit the interpretation of the cecal microbiota results.

Ruminococcaceae is a typical bacterial family involved in carbohydrate metabolism in the animal gut, primarily including genera such as *Faecalibacterium*, *Ruminococcus*, and *Negativibacillus*. Research has shown that *Faecalibacterium* is one of the most abundant endogenous bacteria in the human and animal intestine, typically accounting for approximately 5% of fecal microbiota [34]. It degrades various complex carbohydrates to produce volatile fatty acids such as acetate, propionate, and butyrate, providing 5–15% of the energy for cecal epithelial cell growth [35]. Additionally, it enhances intestinal anti-inflammatory and immunomodulatory functions in animals [36]. Currently, *Faecalibacterium prausnitzii* has been widely investigated as a next-generation probiotic and a biomarker for gut microbiota health [37].

The role of Lachnospiraceae in the animal gut remains controversial [38]. On one hand, similar to Ruminococcaceae, it plays an important role in intestinal health and immune function by degrading various complex polysaccharides to produce volatile fatty acids, making it a major producer of these compounds in the gut [39,40]. On the other hand, it has been associated with the occurrence of various intestinal and extra-intestinal diseases [41]. Whether the significant reduction in the abundance of *Mediterraneibacter*, *Blautia*, and *unclassified_f_Lachnospiraceae* within the Lachnospiraceae family is linked to the marked increase in the abundance of *Faecalibacterium* from the Ruminococcaceae family in this study requires further experimental investigation to elucidate.

Lactobacillaceae is one of the most well-known beneficial bacteria in the animal intestine. It degrades monosaccharides to produce lactate and acetate, significantly lowering the pH of the intestinal microenvironment, thereby promoting gut health and preventing colonization by pathogens [42]. Based on extensive experimental results and long-term practical experience, *Lactobacillus* is one of the few generally recognized as safe bacteria and is a key genus for intestinal health [43]. In this trial, the relatively stable abundance of *Lactobacillus* and *Limosilactobacillus* provided a strong guarantee for the health and growth of the chicks. A study by Pang et al. found that FSBM reduced the abundance of certain harmful bacteria and increased beneficial bacteria [44], which is consistent with our findings.

## 5. Conclusions

In this study, SBM was successfully fermented using *B. subtilis* M2, with the viable count in the M2-FSBM product stabilized at 1.1 × 10^9^ CFU/g. Dietary replacement of SBM with M2-FSBM significantly improved the growth performance of chickens, with earlier introduction and higher substitution rates yielding more pronounced benefits. The growth-promoting effect of M2-FSBM was not attributable to improved feed conversion efficiency, but rather to a significant increase in feed intake. In addition, this effect was associated with a marked increase in small intestinal VH, a significantly elevated VH/CD ratio in the jejunum, and a substantial increase in the abundance of *Faecalibacterium* in the gut, while the abundance of *Lactobacillus* remained relatively stable.

## Figures and Tables

**Table 1:** Composition of basal diet and treatments.

Items	Treatments			
	Con	FSBM25	FSBM50	FSBM75
Ingredient composition (%)				
Corn grain	60.00	60.00	60.00	60.00
SBM	35.00	26.25	17.50	8.75
M2-FSBM	0.00	8.75	17.50	26.25
Soybean oil	1.00	1.00	1.00	1.00
Trace protein-mineral salt ^1^	4.00	4.00	4.00	4.00
Chemical composition (% DM basis)				
Crude protein ^2^	20.48	21.00	21.53	22.06
Calcium ^2^	1.07	1.09	1.11	1.13
Total phosphorus ^2^	0.70	0.68	0.67	0.65
Methionine ^3^	0.46	0.46	0.47	0.47
Metabolizable energy (MJ/kg) ^3^	11.95	11.99	12.04	12.08

Note: Con, control group; FSBM25, 25% M2-FSBM substitution group; FSBM50, 50% M2-FSBM substitution group; FSBM75, 75% M2-FSBM substitution group; SBM, soybean meal; M2-FSBM, *B. subtilis* M2-fermented SBM; DM, dry matter; ^1^ Trace protein-mineral salt was composed of copper sulfate 0.5 g, ferrous sulfate 5.6 g, zinc sulfate 7.5 g, manganese sulfate 8.3 g, sodium selenite 0.1 g, calcium iodate 0.1 g, methionine 32.5 g, vitamin premix 7.5 g, sodium chloride 100.0 g, dicalcium phosphate 312.5 g, limestone 400.0 g, zeolite powder 125.4 g per kg. ^2^ The contents of crude protein, calcium and total phosphorus are calculated based on the measured values obtained from SBM and M2-FSBM assays. ^3^ The contents of methionine and metabolizable energy are calculated based on the Tables of Feed Composition and Nutritive Values in China (2021 thirty-second edition).

**Table 2:** Nutrient contents of SBM and M2-FSBM (*n* = 3).

Items	SBM (%)	M2-FSBM (%)	SEM	*p*-Value
Crude protein	44.79 ± 0.25	50.82 ± 0.75	1.353	0.013
Crude fat	1.18 ± 0.27	1.18 ± 0.04	0.033	0.967
Crude ash	6.35 ± 0.07	6.62 ± 0.10	0.067	0.018
Crude fiber	7.10 ± 0.01	6.58 ± 0.01	0.003	0.509

Note: SBM, soybean meal; M2-FSBM, *B. subtilis* M2-fermented SBM; SEM, standard error of the means; values are expressed as mean ± SD (standard deviation). Within the same row, *p* < 0.05 was considered as a significant difference.

**Table 3:** The effects of partial replacement of SBM by M2-FSBM on growth performance in chickens.

Item	Con	FSBM25	FSBM50	FSBM75	SEM	*p*-Value
Days on test	45	45	45	45		
Replicates	6	6	6	6		
Live weight (g)						
Initial	30.96 ± 0.41	30.94 ± 0.36	30.98 ± 0.20	31.11 ± 0.24	0.061	0.773
15 d	78.78 ± 2.25 ^a^	88.90 ± 5.13 ^b^	88.65 ± 3.15 ^b^	91.77 ± 8.16 ^b^	1.424	0.002
30 d	196.36 ± 13.86 ^a^	207.32 ± 12.23 ^ab^	219.54 ± 18.10 ^bc^	227.2 ± 7.96 ^c^	3.556	0.004
45 d	400.49 ± 26.56 ^a^	421.49 ± 27.36 ^a^	428.90 ± 30.90 ^ab^	453.18 ± 10.74 ^b^	6.188	0.014
Average daily gain (g)						
1–15 d	3.19 ± 0.15 ^a^	3.86 ± 0.34 ^b^	3.84 ± 0.21 ^b^	4.05 ± 0.54 ^b^	0.095	0.002
16–30 d	7.84 ± 0.82 ^a^	7.89 ± 0.64 ^a^	8.73 ± 1.01 ^ab^	9.03 ± 0.30 ^b^	0.189	0.044
31–45 d	13.56 ± 0.96 ^a^	14.28 ± 1.54 ^a^	13.90 ± 0.88 ^a^	15.07 ± 0.66 ^b^	0.231	0.137
1–45 d	8.21 ± 0.58 ^a^	8.687 ± 0.61 ^a^	8.84 ± 0.68 ^a^	9.38 ± 0.24 ^b^	0.137	0.014
Average daily feed intake (g)						
1–15 d	10.39 ± 0.50 ^a^	12.24 ± 0.56 ^b^	12.22 ± 0.24 ^b^	11.97 ± 0.49 ^b^	0.182	<0.001
16–30 d	28.86 ± 1.31 ^a^	31.05 ± 1.60 ^b^	33.26 ± 0.78 ^c^	33.03 ± 1.23 ^c^	0.442	<0.001
31–45 d	47.84 ± 2.76 ^a^	48.74 ± 2.91 ^a^	52.52 ± 2.78 ^b^	54.51 ± 0.48 ^b^	0.735	<0.001
1–45 d	29.03 ± 1.45 ^a^	30.68 ± 1.61 ^b^	32.67 ± 1.25 ^c^	33.17 ± 0.65 ^c^	0.423	<0.001
Feed conversion ratio						
1–15 d	3.27 ± 0.20	3.2 ± 0.35	3.18 ± 0.12	2.99 ± 0.32	0.054	0.359
16–30 d	3.72 ± 0.46	3.95 ± 0.34	3.85 ± 0.39	3.69 ± 0.44	0.081	0.670
31–45 d	3.54 ± 0.35	3.43 ± 0.27	3.79 ± 0.30	3.62 ± 0.15	0.059	0.219
1–45 d	3.55 ± 0.33	3.54 ± 0.23	3.71 ± 0.28	3.54 ± 0.14	0.051	0.605

Note: SBM, soybean meal; M2-FSBM, *B. subtilis* M2-fermented SBM; Con, control group; FSBM25, 25% M2-FSBM substitution group; FSBM50, 50% M2-FSBM substitution group; FSBM75, 75% M2-FSBM substitution group; SEM, standard error of the means. Values are expressed as mean ± SD (standard deviation). Within the same row, values with different superscript letters denote significant differences (*p* < 0.05).

**Table 4:** Histological characteristics of small intestine in chickens (*n* = 6).

Intestine Segments	Items	Con	FSBM75	SEM	*p*-Value
Duodenum	VH (μm)	245.40 ± 17.48 ^a^	301.31 ± 26.03 ^b^	10.41	0.001
	CD (μm)	29.28 ± 3.57	38.99 ± 11.90	2.83	0.084
	VH/CD	7.87 ± 1.58	8.19 ± 1.94	0.49	0.765
Jejunum	VH (μm)	154.07 ± 12.41 ^a^	195.52 ± 18.01 ^b^	7.56	0.001
	CD (μm)	24.68 ± 2.51	25.14 ± 5.38	1.16	0.851
	VH/CD	6.26 ± 0.33 ^a^	8.02 ± 1.58 ^b^	0.41	0.023
Ileum	VH (μm)	142.78 ± 13.65 ^a^	169.67 ± 15.31 ^b^	5.69	0.009
	CD (μm)	22.61 ± 4.34	24.11 ± 5.98	1.46	0.630
	VH/CD	6.44 ± 0.88	7.35 ± 1.74	0.40	0.279

Note: Con, control group; FSBM75, 75% M2-FSBM substitution group; SEM, standard error of the means; VH, Villus height; CD, crypt depth. Within the same row, values with different superscript letters denote significant differences (*p* < 0.05). Values are expressed as mean ± SD.

**Table 5:** Alpha diversity analysis of microbial composition in the cecum of chickens (*n* = 3).

Index	Con	FSBM75	SEM	*p*-Value
Chao1	813.33 ± 57.50	943.33 ± 109.15	43.12	0.13
Simpson (×10^−2^)	1.42 ± 0.10 ^a^	2.23 ± 0.35 ^b^	0.21	0.02
Shannon	7.17 ± 0.03	7.03 ± 0.19	0.06	0.29
ACE	813.33 ± 57.50	943.33 ± 109.15	43.12	0.13

Note: Con, control group; FSBM75, 75% M2-FSBM substitution group; SEM, standard error of the means. Within the same row, values with different superscript letters denote significant differences (*p* < 0.05). Values are expressed as mean ± SD.

**Table 6:** Relative abundance of cecal microbiota at the phylum level in chickens (*n* = 3).

Phylum	Con (%)	FSBM75 (%)	SEM	*p*-Value
Bacillota	97.93	97.86	0.10	0.780
Pseudomonadota	1.73	2.05	0.11	0.188
Actinomycetota	0.24	0.04	0.05	0.014
Cyanobacteria	0.05	ND	0.01	0.026

Note: Con, control group; FSBM75, 75% M2-FSBM substitution group; SEM, standard error of the means. ND indicates that no value was detected.

**Table 7:** Relative abundance of cecal microbiota at the genus level in chickens (*n* = 3).

Genus	Con (%)	FSBM75 (%)	SEM	*p*-Value
*unclassified_f_Lachnospiraceae*	43.71	30.75	0.0294	0.005
*Faecalibacterium*	7.17	24.56	0.0396	0.004
*Incertae_Sedis*	7.34	16.58	0.0210	0.005
*Mediterraneibacter*	9.03	2.58	0.0145	<0.001
*Lactobacillus*	3.63	3.84	0.0015	0.555
*Blautia*	5.52	1.55	0.0089	<0.001
*unclassified_f_Ruminococcaceae*	1.92	1.85	0.0004	0.5681
*Limosilactobacillus*	1.53	1.36	0.0007	0.2523
*Negativibacillus*	1.34	1.51	0.0007	0.2318
others	18.8	15.4	0.0099	0.134

Note: Con, control group; FSBM75, 75% M2-FSBM substitution group; SEM, standard error of the means.

## Data Availability

The raw 16S rDNA sequences presented in this study are openly available in the National Center for Biotechnology Information (NCBI) Sequence Read Archive under accession number PRJNA1306353.

## Supplementary Materials

The following supporting information can be downloaded at https://www.mdpi.com/article/10.3390/microorganisms14071589/s1, Table S1: Serum concentrations of IGF and EGF in chickens (*n* = 6); Table S2: Relative abundance of cecal microbiota at the family level in chickens.
