# Supplementary material for: Bacillus subtilis M2-Fermented Soybean Meal Improves Growth Performance and Modulates Cecal Microbiota in Dongxiang Green-Shell Laying-Type Chicken
Source: Microorganisms. 2026 Jul 21;14(7):1589. doi: 10.3390/microorganisms14071589 (PMC13413807; doi:10.3390/microorganisms14071589)
Supplement: Supplementary file 1 [file microorganisms-14-01589-s001.zip › microorganisms-4382004-supplementary.pdf]

**Supplementary File**

Table S1. Serum concentrations of IGF and EGF in chickens (*n* = 6)

| Growth factor | Birds age | Con | FSBM25 | FSBM50 | FSBM75 | SEM | *p*-value |
|---|---|---|---|---|---|---|---|
| **IGF** (ng/mL) | 15d | 172.26±11.56$^{c}$ | 87.09±11.40$^{a}$ | 169.67±9.46$^{c}$ | 121.25±9.71$^{b}$ | 7.68 | < 0.001 |
| | 30d | 177.10±20.06$^{c}$ | 133.81±11.38$^{b}$ | 99.96±19.21$^{a}$ | 168.38±11.97$^{c}$ | 7.07 | < 0.001 |
| | 45d | 146.42±14.12$^{b}$ | 148.21±15.23$^{b}$ | 83.00±18.36$^{a}$ | 79.67±11.67$^{a}$ | 7.46 | < 0.001 |
| **EGF** (pg/mL) | 15d | 316.31±23.47$^{b}$ | 458.27±38.93$^{c}$ | 440.61±41.66$^{c}$ | 229.69±41.07$^{a}$ | 20.75 | < 0.001 |
| | 30d | 438.87±36.87$^{b}$ | 327.93±31.62$^{a}$ | 454.49±54.62$^{b}$ | 542.59±31.10$^{c}$ | 17.62 | < 0.001 |
| | 45d | 472.43±39.62$^{b}$ | 279.51±31.84$^{a}$ | 513.43±36.33$^{c}$ | 459.39±51.78$^{b}$ | 20.26 | < 0.001 |

Note: IGF, insulin-like growth factor; EGF, epidermal growth factor; Con, control group; FSBM25, 25% M2-FSBM substitution group; FSBM50, 50% M2-FSBM substitution group; FSBM75, 75% M2-FSBM substitution group; SEM, standard error of the means. Within the same row, values with different superscript letters denote significant difference between groups ($P < 0.05$). Values are expressed as mean ± SD.

Table S2. Relative abundance of cecal microbiota at the family level in chickens (*n* = 3)

| Family | Con (%) | FSBM75 (%) | SEM | *p*-Value |
|---|---|---|---|---|
| Lachnospiraceae | 61.54±0.52 | 36.79±2.47 | 5.57 | < 0.001 |
| Ruminococcaceae | 15.34±0.23 | 30.60±2.73 | 3.49 | 0.001 |
| Incertae_Sedis | 5.34±0.32 | 15.63±1.36 | 2.33 | < 0.001 |
| Lactobacillaceae | 5.20±0.37 | 5.25±0.67 | 0.20 | 0.905 |
| Oscillospiraceae | 2.33±0.30 | 3.32±0.60 | 0.28 | 0.065 |
| Others | 10.24±0.16 | 8.40±1.72 | 0.61 | 0.138 |

Note: Con, control group; FSBM75, 75% M2-FSBM substitution group; SEM, standard error of the means. Values are expressed as mean ± SD.
